# A scoping review: Screening questionnaires for identifying tanning addiction

**DOI:** 10.1002/cesm.12092

**Published:** 2024-06-27

**Authors:** John Meisenheimer, Michelle Sobotka, Ronald Yang, Robert P. Dellavalle

**Affiliations:** ^1^ Department of Internal Medicine, Morsani College of Medicine University of South Florida Tampa Florida USA; ^2^ Midwestern University Arizona College of Osteopathic Medicine Glendale Arizona USA; ^3^ University of Colorado Anschutz Medical Campus Denver Colorado USA; ^4^ Department of Veterans Affairs Rocky Mountain Regional Medical Center Aurora Colorado USA

**Keywords:** behavioral addiction, preventative medicine, psychodermatology, screening questionnaires, tanning addiction, UV addiction

## Abstract

**Introduction:**

There is a growing body of evidence that ultraviolet (UV) tanning, whether practiced in indoor tanning salons or outdoors in the sun, is not only linked to detrimental health outcomes but is also addictive through both psychological and physiological mechanisms. In clinical practice, it can be challenging to determine which patients will continue tanning despite being at high risk for developing skin cancer. Our study seeks to identify all available screening questionnaires for tanning addiction that could be used in clinical practice and report on published measures of validity for each screening questionnaire.

**Methods:**

An exhaustive literature search of EMBASE, PubMed, PsycINFO, and Scopus was performed using search criteria including the concepts “UV” and “Addiction.” The most recent search was performed in March 2024 and included all articles from database inception to the time of the search. Studies were included if they reported on screening questionnaires for UV addiction. Articles were excluded from the study if they did not report primary data or did not report on measures of questionnaire validity. Methodology was created using best practices for scoping reviews.

**Results:**

After identifying 171 articles, 106 articles underwent full‐text review, and 26 were included in data extraction. We identified nine questionnaires for tanning addiction, with the modified Cut‐down, Annoyed, Guilty, Eye‐opener (mCAGE), and modified Diagnostic and Statistical Manual of Mental Disorders (mDSM) being most frequently reported on, and the Behavioral Addiction Indoor Tanning Screener (BAITS) being the most promising for future use.

**Conclusions:**

This information should be used to choose questionnaires to be studied against a “gold‐standard” of a panel of psychologists. After defining accuracy of diagnostic tests, studies can be designed to examine interventions for treating tanning addiction, so at‐risk patients can receive specialized therapy, reducing the overall burden of skin cancers.

## 1 INTRODUCTION

Ultraviolet (UV) tanning, whether practiced in indoor tanning salons or outdoors in the sun, is linked to a range of detrimental health outcomes. According to a meta‐analysis of 27 observational studies, users of indoor tanning devices had a 20% increased risk of melanoma, which doubled if tanning started under the age of 35 [1]. UV radiation penetrates the skin, damaging its cellular structure at the DNA level and increasing the risk of skin cancers, including non‐melanoma skin cancers [2, 3, 4]. Furthermore, prolonged UV exposure accelerates the skin's aging process, leading to premature wrinkles, burns, and other visible signs of skin damage [5]. The widespread accessibility of tanning options, including easily accessible gyms and spas, has exacerbated these issues, normalizing practices that are inherently harmful [6].

Despite evidence regarding the harmful consequences of tanning, people continue to engage in tanning practices. According to a qualitative study, participants who regularly underwent tanning understood its associated health risks, yet the participants continued to tan with the goal of enhancing their appearance. Multiple participants also mentioned how difficult it was to stop tanning, with multiple failed attempts and withdrawal symptoms, such as feeling ill [2]. A study by Miller et al. found that 7% of evaluated 11th grade students met criteria for tanning addiction [7]. With increasing evidence suggesting that UV‐seeking behavior has addictive features, the importance of elucidating the mechanism of this phenomenon becomes apparent.

Several studies offer biologic explanations for the physiology underlying addiction to UV radiation. Fell et al. [8] conducted a study on rodents and suggested that repeated UV exposure increases circulating beta‐endorphins, inducing an opioid receptor‐mediated addiction. They found that this mechanism elevated pain‐related thresholds, with opioid blockade by naloxone causing withdrawal signs such as teeth chattering and bouts of self‐grooming. These UV‐induced nociceptive and behavioral effects were absent in mice with the beta‐endorphin gene knockout in epidermal keratinocytes [8].

Multiple studies on human participants suggest that UV light exposure activates neurochemical pathways in the brain that reinforce tanning behavior. Several studies document that UV light has reinforcing properties: UV radiation exposure leads to the release of endogenous opioids and dopamine, and tanning sessions increase blood flow to brain areas associated with the drug‐induced reward pathways [9, 10, 11, 12]. UV‐induced production of neurochemicals including dopamine and endorphins lead to increased relaxation and decreased pain, establishing a reinforcing cycle that perpetuates the desire for more tanning sessions.

Genetics also play a role in susceptibility to UV radiation addiction, with certain individuals being more prone to the rewarding effects of UV exposure and the associated endorphin release. A study by Sanna et al., [13] using data from twins, suggests that the repeated desire to spend time in the sun is significantly heritable. They also identify five genes previously associated with addiction in individuals with sun‐seeking behavior [13]. Flores et al. [14] implemented a case‐control study to investigate how single‐nucleotide polymorphisms (SNPs) can affect dopamine reward pathways in the brain to facilitate tanning addiction. They found that some SNPs were associated with an increased likelihood of using indoor tanning beds [14]. This physiological dependence can turn tanning into an addictive behavior that is hard to stop.

Delineating frequent use from dependence can be difficult, as there is an overlap between the two concepts [15]. A subset of patients show signs of addiction (psychological dependence) and dependence (physiologic dependence) [15]. These patients find themselves trapped in a cycle of compulsive behavior with difficulty quitting, feelings of guilt when tanning too much, and tanning that becomes physically hazardous [16]. One of the benefits of differentiating between overuse and dependence is targeting those who may be more resistant to change when faced with consequences of their use [15]. The “gold standard” for identifying dependence is typically a determination made by a panel of psychologists after a battery of interviews [17]. Clinically it is more practical to use screening questionnaires as a high‐sensitivity tool for identifying those at risk for dependence.

Due to the growing evidence of a physiologic and psychologic basis for UV dependence, screening questionnaires have been created to differentiate between individuals who are frequent tanners and those who are addicted to tanning. It can be difficult to determine the validity of each of these screening questionnaires, and which ones may be best employed for identifying tanning addiction. At the time of this review, there are no studies that use a “gold standard” test as a comparator, further complicating comparisons between the questionnaires. Still, there are methods for examining the validity of the questionnaires beyond comparison with a “gold standard.”

This leads to the question, what are the current screening questionnaires for identifying UV addiction and how has the validity of these questionnaires been assessed? Here we have identified and reviewed the various screening questionnaires that have been reported on in the literature for identifying tanning dependence. We also attempt to make comparisons between each of the studies, the strengths, and weaknesses as well as the potential for utilization in a clinical setting.

## 2 METHODS

**Figure 1:**
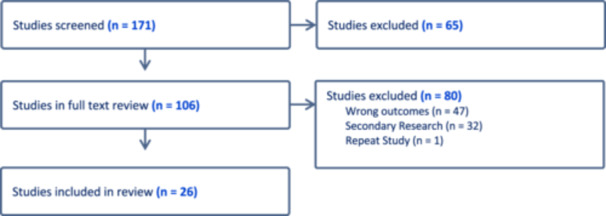
Prisma style flow sheet of the article screening process.

We followed the methodology and practices for conducting scoping reviews created by Arksey and O'Malley [18]. Per their methodology, certain aspects of the study protocol were developed post hoc; for example, inclusion and exclusion criteria were defined following the initial literature search, and the data extraction template was created following full‐text review. A Preferred Reporting Items for Systematic Reviews and Meta‐Analyses extension for Scoping Reviews (PRISMA‐ScR) checklist was completed and submitted with the review. An a priori study protocol was not published before undertaking the scoping review. An exhaustive literature search was performed of the databases EMBASE and PubMed. We employed search criteria including the concepts “UV” and “Addiction.” Search strategies were reviewed by an information specialist at the University South Florida, PsycINFO and Scopus were retrospectively added into the search strategy. The most recent search was performed in March 2024 and included all articles from database inception to the time of the search. The full search criteria we employed are included as a supplement. We used COVIDENCE for the screening and full‐text review. Two reviewers (JM and MS) participated in the abstract screening identifying articles that were relevant to the topic of UV addiction. Discrepancies between reviewers at abstract screening were handled through consensus meeting between reviewers, with a low threshold to include articles for full‐text review. Following this, two reviewers (JM and MS) screened full‐text articles in their entirety for inclusion in the data extraction and analysis. Inclusion criteria for data extraction were defined broadly. We did not limit included studies by population characteristics nor did we limit included studies by context. All geographic areas, settings, and languages were included. Conceptually included studies needed to report results on questionnaires designed for identifying UV addiction. Articles were excluded if they did not include primary data or report results that allowed for analysis of some component of validity for the employed screening questionnaires. Figure 1 shows a visual representation of our article screening process. Following full‐text review, a data extraction template was created in Excel and approved by two authors (JM and MS). Data was extracted by a single author with a second author reviewing all extracted data from included articles for completeness using the extraction template with any concerns being handled through consensus between both authors.

In the absence of a “gold standard” by which to make comparisons, screening questionnaires can be graded on various subjective and objective criteria that allow for determination of different aspects of validity [19]. The extraction sheet was designed to extract reported data on questionnaire validity, of which there are multiple subtypes. We extracted data on face validity, a subjective measure that examines whether questions ask about things one would expect it to ask about [19]. Data on content validity, a subjective assessment of whether all the aspects of a concept are covered, was also extracted [19]. Data on construct validity was extracted as a correlation of questionnaire results to other related variables [19]. This included comparing the correlation between the results of two questionnaires or how a questionnaire correlated with a person's tanning frequency, for example. Correlation between questionnaire outcomes and substance abuse, behavioral addictions, body dysmorphic disorder, age at first tan, and various psychiatric comorbidities could be considered a component of construct validity. We did not extract data on these factors to limit the amount of extracted information, as they were deemed to be of less relevance.

Internal consistency, a measure of how related each question in the questionnaire is to the other questions being asked was extracted using Cronbach's alpha and related variables. Test‐retest reliability was extracted by measures of whether participants score similarly on a repeated test at a later timepoint [19].

All extracted data was synthesized into two tables reporting study characteristics and reported measures of validity by one author with review by a second author to ensure all extracted data was reported. A single author further summarized reported characteristics and measures of validity deemed most important to the research question.

## 3 RESULTS

Our search resulted in 171 articles for abstract review. Of these, 106 articles underwent full‐text review with 26 articles being included for data extraction. Table 1 shows the study characteristics of the included articles. Most study populations were predominately female; five study populations were entirely women and one was entirely men. The average age for studies tended to be younger; the oldest study population had an average age of 38 with the age range across all studies being 14–54.

**Table 1:** Condensed demographic and study design for included articles.

Author (year)	Country	Study design	Participants	Data collection period	Percent male	Percent female	Age range of participants	Average age of participants	Setting	Format	Indoor vs. outdoor tanning
Ashrafioun (2014)	USA	Cross‐sectional	533	Fall semester 2011	28%	72%	Not Reported	19.5 (1.7)	Undergraduates in psychology coursses from a large public midwestern university	Web based	Indoor
Adniof (2014)	USA	Case‐control	20	Not Reported	Not Reported	Not Reported	Not Reported	Not Reported	Not Reported	In person	Indoor
Venning (2020)	Australia	Cross‐sectional	485	2018	Not Reported	Not Reported	Not Reported	Not Reported	Individuals at a skin exam	Not Reported	Not Reported
Warthan (2005)	USA	Cross‐sectional	145	July 2002	32%	68%	18–53	Not Reported	Beach Goers in Galveston Island, Texas	Not Reported	Both
Toledo (2019)	Finland	Cross‐sectional	229	July–August 2015	16%	84%	Not Reported	Not Reported	Finish speaking adults at beaches and in parks in Tampere and Pori, Finland	Half web‐based and half paper	Outdoor
Stawczyk (2011)	Poland	Cross‐sectional	496	January‐March 2010	0%	100%	15–30	21.2 (2.6)	University students in large Midwestern United States city	Web‐based and in person	Both
Stapleton (2016)	USA	Cross‐sectional	164	October‐November, April‐May (year unclear)	17.70%	82.30%	Not Reported	20.1 (2.0)	Undergraduate students attending southeastern United States University	Web based	Indoor
Schneider (2015)	Germany	Cross‐sectional	60	Not Reported	Not Reported	Not Reported	14–45	Not Reported	Germany	Computer aided telephone interviews	Indoor
Reed (2016)	USA	Cross‐sectional	93	Not Reported	0%	100%	18–36	20.19 (2.87)	Undergraduates in introductory psychology course	Not Reported	Indoor
Poorsattar (2007)	USA	Cross‐sectional	375	December 2005 and January 2006	35%	65%	17–30	Not Reported	Undergraduates at University of Washington in Seattle	Paper	Both
Andreassen (2018)	Norway	Cross‐sectional	23537	March ‐ May 2014	35%	65%	16–88	35.8 (13.3)	Norwegians	Web based	Not Reported
Ashrafioun (2015)	USA	Cross‐sectional	421	November and December (year unclear)	27%	73%	Not Reported	19.5 (1.8)	Undergraduate students in psychology courses at a public midwestern university	Web based	Both
Ashrafioun (2014)	USA	Cross‐sectional	414	Not Reported	27%	73%	Not Reported	19.5 (1.8)	Students from a large midwestern United States university	Web based	Both
Becirevic (2017)	USA	Cross‐sectional	80	Not Reported	0%	100%	Not Reported	19.55 (2.52)	Introductory‐level undergraduate psychiatry course	Web based	Indoor
Cartmel (2017)	USA	Cross‐sectional	499	Not Reported	25%	75%	Not Reported	38.5 (4.8)	From case control study of early onset basal cell carcinoma in conneticut	Web based	Both
Cartmel (2013)	USA	Cross‐sectional	178	Not Reported	14%	86%	Not Reported	Not Reported	Patients diagnosed with basal cell carcinoma before age 40 in conneticut	Web based	Indoor
Mosher (2010)	USA	Cross‐sectional	229	September to December 2006	31.60%	67.50%	Not Reported	Not Reported	Undergraduates from a state university in north eastern United States	Paper	Indoor
Miller (2018)	USA	Cross‐sectional	2637	2013	45%	55%	Not Reported	16.06 (0.43)	Public high schools in los Angeles	Not Reported	Not Reported
Mays (2020)	USA	Cross‐sectional	389	September to December 2016	0%	100%	18–30	Not Reported	Community sourounding a large cancer research center	Web based	Indoor
Hillhouse (2012)	USA	Longitudinal, cross‐sectional	296	October 1, 2008 ‐ May 31, 2009	35.50%	64.50%	Not Reported	21.8 (5.85)	College age students at East Tennessee State University	Not Reported	Indoor
Heckman (2014)	USA	Cross‐sectional	306	2009‐2011	0%	100%	18–25	19.9 (1.6)	Psychology students at a northeastern university	Web based	Indoor
Heckman (2008)	USA	Cross‐sectional	400	Spring semester 2006	25%	75%	Not Reported	21 (5.42)	Southwestern metropolitan university community	Web based	Both
Diehl (2018)	Germany	Cross‐sectional	883	Pretest August 2015, survey October‐December 2015	Not Reported	Not Reported	14‐45	Not Reported	German Residents	Telephone	Indoor
Nogg (2018)	USA	Cross‐sectional	230	2016	100%	0%	15–35	24.66 (5.44)	Sexual minority males in San Diego, California	Web based	Indoor
Banerjee (2014)	USA	Cross‐sectional	551	2011	36.10%	63.90%	18–25	19.98 (1.23)	Students in introductory communication courses at a North‐Eastern University	Paper	Both
Benet (2018)	USA	Cross‐sectional	102	Not Reported	25.50%	74.50%	Not Reported	22.5	University students in large Midwestern United States city	Not reported	Both

We identified nine different screening questionnaires for evaluating tanning addiction: modified Cut‐down, Annoyed, Guilty, Eye‐opener (mCAGE), Modified Diagnostic and Statistical Manual of Mental Disorders (mDSM), Structured Interview for Tanning Abuse and Dependence (SITAD), Behavioral Addiction Indoor Tanning Screener (BAITS), Craving to Tan Questionnaire (CTQ), Bergin Tanning Addiction Scale (BTAS), Tanning Problem Index (TPI), Tanning Pathology Scale (TAPS) and Tanning Passion Scale (TPS). Supporting Information S1 and S2 shows the data extracted from each of the studies.

Of the questionnaires, the mCAGE and mDSM were the most frequently studied, with some aspect of validity being examined in 16 and 11 studies, respectively. The frequency of the studied questionaires is recorded in Table 2. Importantly, the ways in which the questionnaires were applied was not consistent across studies, with slight variations in the wording of questions, mainly for studies of mDSM and mCAGE. The mDSM was the least consistent across all studies. Additionally, some studies reported a combined outcome of mCAGE and mDSM, but even within that group there was variation with some using a combined outcome of positivity on either mCAGE or mDSM as criteria for tanning addiction, and others using positivity on both mCAGE and mDSM as criteria for tanning addiction.

**Table 2:** Matrix of screening questionnaires examined by included articles.

Author (year)	mCAGE	mDSM	BTAS	SITAD	CTQ	BAITS	TPI	TAPS	TPS
Ashrafioun (2014)	x	x							
Adniof (2014)		x							
Venning (2020)			x						
Warthan (2005)	x	x							
Toledo (2019)				x					
Stawczyk (2011)	x								
Stapleton (2016)				x		x			
Schneider (2015)	x								
Reed (2016)	x	x							
Poorsattar (2007)	x								
Andreassen (2018)			x						
Ashrafioun (2015)	x	x			x		x		x
Ashrafioun (2014)		x			x		x		
Becirevic (2017)						x			
Cartmel (2017)	x	x							
Cartmel (2013)	x								
Mosher (2010)	x	x							
Miller (2018)	x								
Mays (2020)	x	x							
Hillhouse (2012)				x					
Heckman (2014)	x	x						x	
Heckman (2008)	x	x							
Diehl (2018)						x			
Nogg (2018)						x			
Banerjee (2014)	x								
Benet (2018)	x								
Total	16	11	2	3	2	4	2	1	1

Abbreviations: BAITS, Behavioral Addiction Indoor Tanning Screener; BTAS, Bergin Tanning Addiction Scale; CTQ, Craving to Tan Questionnaire; mCAGE, modified Cut‐down, Annoyed, Guilty, Eye‐opener; mDSM, Modified Diagnostic and Statistical Manual of Mental Disorders; TAPS, Tanning Pathology Scale; TPI, Tanning Problem Index; TPS, Tanning Passion Scale; SITAD, Structured Interview for Tanning Abuse and Dependence.

In terms of internal consistency, BTAS had the highest reported value of 0.94, but reports this value as an Omega value, rather than Cronbach's alpha. Omega tends to give slightly higher estimates than Cronbach's alpha, but the difference is usually small, particularly as the value increases. CTQ has the highest reported alpha values ranging from 0.86 to 0.91. mCAGE and mDSM consistently had the lowest values, ranging from 0.38–0.71 to 0.52–0.62, respectively. When mCAGE and mDSM are treated as a combined outcome, the measure improves with a range of 0.69–0.70. The lowest alpha values were obtained from studies completed in languages other than English, and with translated questionnaires.

The most commonly reported measures were evaluations of construct validity, although it was not always explicitly stated as such. Overall, there was a significant difference in tanning frequency for addicted versus not addicted participants identified by all the questionnaires. A study using mCAGE compared the rates of tanning after BCC diagnosis and found that although a higher percentage of the group tanning after diagnosis met criteria for mCAGE, there was not a significant difference between the two groups. Additionally, many studies compare scores between multiple questionnaires, and in general found statistically significant agreement across questionnaires in identifying participants addicted to tanning.

Only one study, examining SITAD, reports on test‐retest reliability, showing 97% agreement after 3 weeks for participants who were identified as tanning dependent. Phi, a measure of reliability for test‐retest calculations, was 0.84 indicating fair reliability of this estimate. Two studies assess how questionnaires were being interpreted by participants through cognitive interviews, which could be considered as an aspect of face validity. These interviews found the mCAGE was sometimes poorly understood, while BAITS did not have any questions that were misunderstood.

Content validity was occasionally assessed. Comparative fit index was reported for BTAS and TAPS, while the Tucker–Lewis Index was reported for just BTAS. Eigen values and factor loadings were given for BAITS, BTAS, TAPS, and CTQ.

## 4 DISCUSSION

Given the heterogeneity of the extracted data, picking the “best” questionnaire is a subjective process. While the mCAGE and mDSM have promising data, there are concerns regarding the internal consistency of these tools. In addition, it is unclear if the wording of the mCAGE and mDSM are the same across all studies we examined. The SITAD has promising data regarding validity but requires a higher time investment than is typical for a simple screening questionnaire and may be more useful as a comparator for use in research on screening questionnaires, as opposed to a tool for use in a clinical setting. Overall, the BAITS seems to be the most promising based on the available data, although it is limited to indoor tanning behaviors. Adjusting the wording to include all tanning behaviors is likely possible, although new studies would be needed to show the validity of an updated measure regarding populations of both indoor and outdoor tanners. Newer questionnaires like BTAS and CTQ also have auspicious data, but are lacking in studies showing construct validity. Ideally, more studies should be completed focusing on test‐retest reliability of all measures, as well as studies on identifying how well questionnaires perform in identifying continued tanning after skin cancer diagnosis.

While our scoping review does have strengths due to its broad and exhaustive scope of the subject matter, there are limitations as well. Many of the studies we reported on focused on age groups or genders, so while our inclusion criteria did not limit to specific populations, certain populations may be over‐represented. Another limitation of the study is in including all languages, particularly in the case of screening questionnaires. As many of these questionnaires are designed for use in English, the process of translation creates inconsistencies in outcomes. Furthermore, we did not make a distinction between indoor and outdoor tanning, and it is unclear if the screening questionnaires work differently depending on these patterns of use. These concessions were ultimately necessary to achieve our goals of fully defining the evidence available for validity of UV addiction screening questionnaires.

Patients who have sun damage and are diagnosed with skin cancer are at higher risk for developing future skin cancers than the general population. This is due to genetic factors, in which people who have already developed one skin cancer are more likely to be genetically predisposed to developing more when compared to the general population, as well as the concept of field cancerization [20]. Field cancerization is based on the idea that cancer does not typically develop after a single mutation but requires multiple mutations in different genes to progress to a cancer. For patients who have extensive sun damage and already developed one skin cancer that has been treated, they have likely accumulated multiple mutations within the area of the remaining normal‐appearing skin, and require less exposure to develop another cancer [20].

For some patients, they may find it easy to discontinue tanning after being diagnosed with a skin cancer and being counseled on the risk of further UV exposure. For others, addiction and dependence to tanning may be a barrier to reducing UV exposure after skin cancer diagnosis. The ability to screen for patients at risk of tanning addiction would help identify patients at high risk for future skin cancers to refer them for psychological or pharmacologic treatment.

Explorations of possible treatments for tanning addiction have already been proposed. Psychotherapy is being explored as a potential treatment option for tanning dependence and is often treated in the same way as other behavioral addictions. The possibility of using naltrexone, a mu‐opioid antagonist used to treat opioid and alcohol use disorders, has also been proposed. This is based on studies showing that tanning addiction may be partially mediated through the induction of beta‐endorphins by UV light, which bind to mu‐opioid receptors, activating reward pathways thus creating a sedating and pleasurable effect. Blocking these receptors could theoretically reduce the physiologic component of UV addiction, allowing for easier treatment of the psychological and behavioral aspects of addiction.

## 5 CONCLUSION

Tanning addiction is a phenomenon with well‐studied physiologic and psychologic basis. From our review, we have identified a wide range of screening tools that could be employed to identify tanning addiction in a clinical setting. While the current studies do examine important aspects of screening questionnaires concerning validity, this information should be used to choose one or multiple questionnaires to be studied against a “gold‐standard” of a panel of psychologists. Once a gold standard is established, future studies can focus on quantifying the consequences of tanning addiction, particularly in subsets of patients at high risk for developing skin cancer. After this has been accomplished, studies should be designed to examine interventions for treating tanning addiction, so at‐risk patients can receive specialized therapy, reducing the overall burden of skin cancers.

## AUTHOR CONTRIBUTIONS

**John Meisenheimer**: Conceptualization; data curation; investigation; methodology; visualization; writing—original draft; writing—review & editing. **Michelle Sobotka**: Data curation; visualization; writing—original draft; writing—review & editing. **Ronald Yang**: Writing—original draft; writing—review & editing. **Robert P Dellavalle**: Resources; supervision.

## PEER REVIEW

The peer review history for this article is available at https://www.webofscience.com/api/gateway/wos/peer-review/10.1002/cesm.12092.

## Supporting information

Supporting information.

Supporting information.

## Data Availability

The data that support the findings of this study are available from the corresponding author upon reasonable request.
