# Supplementary material for: A scoping review: Screening questionnaires for identifying tanning addiction
Source: Cochrane Evid Synth Methods. 2024 Jun 27;2(7):e12092. doi: 10.1002/cesm.12092 (PMC11795949; doi:10.1002/cesm.12092)
Supplement: Supplementary file 1 — Supporting information. [file CESM-2-e12092-s001.docx]

| PubMed |  |  |
| --- | --- | --- |
| *Concepts* | UV | addiction |
| Entry Terms and Keywords  (Title/Abstract) | “Ultraviolet Ray*”[tiab] OR  “Ultra Violet Ray*”[tiab] OR  “UV Light”[tiab] OR  “Actinic Ray*”[tiab] OR  “Ultraviolet Light*”[tiab] OR  “UV Radiation*”[tiab] OR  “Ultraviolet Radiation*”[tiab] OR  “Ultraviolet Black Light*”[tiab] OR  Sunshine[tiab] OR  Sunlight[tiab] OR  “sun shine”[tiab] OR  “sun light”[tiab] OR  “sun exposure*”[tiab] OR  “UV exposure*”[tiab] OR  “sunburn”[tiab] OR  “sunburn”[tiab] OR  “Tanning”[tiab] OR  “Tan”[tiab] | Addictive[tiab] OR  Addiction[tiab] OR  Addicted[tiab] OR |
| MeSH  (Medical Subject Headings) | "Ultraviolet Rays"[Mesh] OR  "Sunlight"[Mesh] OR  "Sunbathing"[Mesh] OR  “Tanning”[Mesh] | "Behavior, Addictive"[Mesh] |

| EMBASE |  |  |
| --- | --- | --- |
| *Concepts* | UV | addiction |
| Entry Terms and Keywords  (Title/Abstract) | “Ultraviolet Ray*”:ti,ab OR  “Ultra Violet Ray*”:ti,ab OR  “UV Light”:ti,ab OR  “Actinic Ray*”:ti,ab OR  “Ultraviolet Light*”:ti,ab OR  “UV Radiation*”:ti,ab OR  “Ultraviolet Radiation*”:ti,ab OR  “Ultraviolet Black Light*”:ti,ab OR  Sunshine:ti,ab OR  Sunlight:ti,ab OR  “sun shine”:ti,ab OR  “sun light”:ti,ab OR  “sun exposure*”:ti,ab OR  “UV exposure*”:ti,ab OR  “Tanning*”:ti,ab OR  “Tan”:ti,ab | Addictive:ti,ab OR  Addiction:ti,ab OR  Addicted:ti,ab |
| EMTREE  (Medical Subject Headings) | 'ultraviolet radiation'/exp OR  'sunbathing'/exp OR  ‘sunlight’/exp  ‘tanning’/exp | 'behavioral addiction'/exp |

| Psycinfo |  |  |
| --- | --- | --- |
| *Concepts* | UV | addiction |
| Entry Terms and Keywords  (Title/Abstract) | AB(“Ultraviolet Ray*” OR  “Ultra Violet Ray*” OR  “UV Light” OR  “Actinic Ray*” OR  “Ultraviolet Light*” OR  “UV Radiation*” OR  “Ultraviolet Radiation*” OR  “Ultraviolet Black Light*” OR  Sunshine OR  Sunlight OR  “sun shine” OR  “sun light” OR  “sun exposure*” OR  “UV exposure*” OR  “sunburn” OR  “sunburn” OR  “Tanning” OR  “Tan”)  OR  TI(“Ultraviolet Ray*” OR  “Ultra Violet Ray*” OR  “UV Light” OR  “Actinic Ray*” OR  “Ultraviolet Light*” OR  “UV Radiation*” OR  “Ultraviolet Radiation*” OR  “Ultraviolet Black Light*” OR  Sunshine OR  Sunlight OR  “sun shine” OR  “sun light” OR  “sun exposure*” OR  “UV exposure*” OR  “sunburn” OR  “sunburn” OR  “Tanning” OR  “Tan”) | AB(“Addictive” OR “Addiction” OR “Addicted”)  OR  TI(“Addictive” OR “Addiction” OR “Addicted”) |
| PsycInfo controlled vocabulary | DE"burns"  OR  DE"Tanning" | DE“Nonsubstance Related Addictions” OR  DE“Nonsubstance Related Addiction Measures”  OR  DE"Online Addiction Measures" |

| Scopus |  |  |
| --- | --- | --- |
| *Concepts* | UV | addiction |
| Entry Terms and Keywords  (Title/Abstract) | Title(“Ultraviolet Ray*” OR  “Ultra Violet Ray*” OR  “UV Light” OR  “Actinic Ray*” OR  “Ultraviolet Light*” OR  “UV Radiation*” OR  “Ultraviolet Radiation*” OR  “Ultraviolet Black Light*” OR  Sunshine OR  Sunlight OR  “sun shine” OR  “sun light” OR  “sun exposure*” OR  “UV exposure*” OR  “sunburn” OR  “sunburn” OR  “Tanning” OR  “Tan”)  OR  ABS(“Ultraviolet Ray*” OR  “Ultra Violet Ray*” OR  “UV Light” OR  “Actinic Ray*” OR  “Ultraviolet Light*” OR  “UV Radiation*” OR  “Ultraviolet Radiation*” OR  “Ultraviolet Black Light*” OR  Sunshine OR  Sunlight OR  “sun shine” OR  “sun light” OR  “sun exposure*” OR  “UV exposure*” OR  “sunburn” OR  “sunburn” OR  “Tanning” OR  “Tan”) | Title(“Addictive” OR “Addiction” OR “Addicted”)  OR  ABS(“Addictive” OR “Addiction” OR “Addicted”) |
