# Supplementary material for: A scoping review: Screening questionnaires for identifying tanning addiction
Source: Cochrane Evid Synth Methods. 2024 Jun 27;2(7):e12092. doi: 10.1002/cesm.12092 (PMC11795949; doi:10.1002/cesm.12092)
Supplement: Supplementary file 2 — Supporting information. [file CESM-2-e12092-s002.pdf]

<table>
<tr><th>First Author</th><th>Study</th><th>Total Population</th><th>Validity</th><th>Internal consistency</th></tr>
<tr>
<td>Lisham Ashrafioun (2014)</td>
<td>Tanning Addiction and Psychopathology: Further Evaluation of Anxiety Disorders and Substance Abuse[1]</td>
<td>533</td>
<td>
Construct Validity
<table>
<tr><th></th><th>Negative mCAGE (n=468)</th><th>Positive mCAGE (n=65)</th></tr>
<tr><td>DSM-IV score (SD)</td><td>1.5 (1.5)*</td><td>3.6 (1.6)*</td></tr>
<tr><td>Days tanning past month (SD)</td><td>3.9 (5.2)*</td><td>11.3 (8.1)*</td></tr>
<tr><td>History of tanning bed burn (percent)</td><td>73 (16%)</td><td>13 (20%)</td></tr>
</table>
<table>
<tr><th></th><th>Negative DSM-IV (n=368)</th><th>Positive DSM-IV (n=165)</th></tr>
<tr><td>DSM-IV score (SD)</td><td></td><td></td></tr>
<tr><td>Days tanning past month (SD)</td><td>2.9 (4.0)*</td><td>8.7 (7.8)*</td></tr>
<tr><td>History of tanning bed burn (percent)</td><td>48 (10%)†</td><td>48 (29%)†</td></tr>
</table>
<table>
<tr><th colspan="2">OR for nonproblematic v. problematic tanning on mCAGE</th><th colspan="2">OR for nondependent v. dependent tanning by DSM-IV</th></tr>
<tr><td>0-1 days tanning in the last 30 days</td><td>Reference group</td><td>0-1 days tanning in the last 30 days</td><td>Reference group</td></tr>
<tr><td>2-8 days tanning in the last 30 days</td><td>4.63 (1.83-11.71)</td><td>2-8 days tanning in the last 30 days</td><td>2.58 (1.49-4.48)</td></tr>
<tr><td>>9 days tanning in the last 30 days</td><td>17.41 (7.07-42.90)</td><td>>9 days tanning in the last 30 days</td><td>8.26 (4.44-15.35)</td></tr>
</table>
</td>
<td>Cronbach's alpha mCAGE = 0.707620882

Tanning-DSM = 0.618983337</td>
</tr>
<tr>
<td>Bryon Adinoff (2014)</td>
<td>Dopamine Efflux in Response to Ultraviolet Radiation in Addicted Sunbed Users[2]</td>
<td>20</td>
<td>
Construct Validity
<table>
<tr><th></th><th>DSM-5 Negative (n=10)</th><th>DSM-5 Positive (n=10)</th></tr>
<tr><td>Lifetime sunbed exposures mean (SD)</td><td>542(±710)</td><td>1851(±1420)</td></tr>
<tr><td>DSM-5 criteria met mean (SD)</td><td>0.5(±0.5)</td><td>5.7(±1.9)</td></tr>
<tr><td>Sham UV light: Change in BPndand lifetime sun exposures/years of tanning linear correlation linear correlation</td><td>Reported as no significant change in BPnd</td><td>Reported as no significant change in BPnd</td></tr>
<tr><td>UV light: Change in BPnd and lifetime sun exposures/years of tanning linear correlation</td><td>r = - 0.05, p = 0.9</td><td>r = -0.752, p = 0.01</td></tr>
</table>
</td>
<td></td>
</tr>
</table>

Victoria L Venning (2020)	Nothing Healthy About a Tan – Increasing Prevalence of Tanning Addiction[3]	485	**Construct Validity**			
				**Average BTAS score for those answering no**	**Average BTAS score for those answering yes**	
			**Do you feel happier and less stressed after tanning?**	2.8*	4.15*	
Molly M. Warthan (2005)	UV Light Tanning as a Type of Substance-Related Disorder[4]	145	**Construct Validity**			
				**Negative DSM-IV-TR**	**Positive DSM-IV-TR**	
			**Negative mCAGE**	56†	51†	
			**Positive mCAGE**	12	26	
				**Negative mCAGE**	**Positive mCAGE**	
			**Likelihood reason for going to the beach is to tan**	Reference group	2.2#	
			**Likelihood to tan more than 2 days a week**	Reference group	Not reported	
				**Negative DSM-IV-TR**	**Positive DSM-IV-TR**	
			**Likelihood reason for going to the beach is to tan**	Reference group	2.2#	
			**Likelihood to tan more than 2 days a week**	Reference group	2.7#	
Anna Toledo (2019)	Tanning Dependence and Seasonal Affective Disorder are Frequent Among Sunbathers but are not Associated[5]	229	**Construct Validity**			
				**Negative SITAD (n=152)**	**SITAD tanning abuser (n=59)**	**SITAD tanning dependent (n=18)**
			**Percent sunbathing frequently whenever positive**	37%‡	75%‡	83%‡
			**Percent having tanned their skin indoors**	40%‡	58%‡	67%‡
			**Percent having ever sunbathed to tan**	91%‡	98%‡	100%‡

Marta Stawczyk (2011)	Evaluation of Risk of Tanning Addiction in a Selected Population of Women Aged 15-30[6]	496	**Construct Validity**			
					**Negative mCAGE (n=342)**	**Positive mCAGE (n=74)**
			**Percent of women who indoor tan who tan for a duration < 8 minutes**		43.40%†	17.20%†
			**Percent of women who indoor tan who tan for a duration 8-10 minutes**		34.70%	34.40%
			**Percent of women who indoor tan who tan for a duration 10-12 minutes**		17.70%†	29.30%†
			**Percent of women who indoor tan who tan for a duration >12 minutes**		3.90%†	18.90%†
			**Percent of women who have not used a sunbed**		26.30%	20.30%
			**Percent of women who have used a sunbed a few times in their life**		43.50%	40.50%
			**Percent of women who use a sunbed less than 10 times a year**		22.20%	24.30%
			**Percent of women who use a sunbed more than 10 times a year**		7.80%†	14.80%†
Jerod L. Stapleton (2016)	The Behavioral Addiction Indoor Tanning Screener (BAITS): An Evaluation of a Brief Measure of Behavioral Addictive Symptoms[7]	164	**Construct Validity**			
				**Endsorsed no BAITS item (n=133)**	**Endorsed 1 BAITS item (n=16)**	**Endorsed >1 BAITS item (n=15)**
			**Percent identified as dependent by SITAD**	1%†	25%†	73%†
			**Subsequent 6-month indoor tanning frequency in mean hours (SD)**	10.15 (15.37)**	18.28 (18.16)**	47.10 (50.92)**

Sven Schneider (2015)	Casting Shadows on the Prevalence of Tanning Dependence: An Assessment of mCAGE Criteria[8]	60	**Construct Validity**			**Cronbach's alpha mCAGE** = 0.38
				**Past users n =1145**	**Current users n = 700**	
			**Positive mCAGE**	11.70%	14.50%	
			**Do you try to cut down on the time you spend on sunbeds?**	28.50%	27.80%	
			**Have people ever criticized your indoor tanning habits and have you been annoyed by that criticism?**	2.60%†	5.00%†	
			**Do you ever feel guilty because of your sunbed usage?**	20.20%	19.90%	
			**Is using a sunbed one of the first things you think about when waking up in the morning?**	2.10%†	7.50%†	
				**Negative mCAGE (n=599)**	**Positive mCAGE (n=101)**	
			**Median (IQR) tanning sessions within last 12 months**	5.4 (2.0–15.0)†	9.7 (3.5–24.0)†	
			**Median (IQR) tanning sessions within last 6 months**	2.0 (1.0–6.0)†	4.0 (1.0–12.0)†	
			**Median (IQR) tanning sessions within last 3 months**	2.0 (0.0–5.0)	3.0 (0.6–6.4)	
			**Median (IQR) tanning sessions within last 1 month**	1.0 (0.0–2.0)	1.7 (0.0–2.0)	
			**Median (IQR) tanning sessions per week**	0.2 (0.1–0.6)†	0.5 (0.2–1.5)†	
			**Objective tolerance measured by percentage of participants with intensification of exposure over time**	11.2†	4.1†	
			**Subjective tolerance measured by percentage of participants with agreement to the question "Do you think you need to continuously increase the time of UV light exposure in order to keep your tan?"**	12.2†	27.3†	
			**Face Validity**<br>15 participants, none with confusion over question 1<br>4 participants with confusion over question 2, with some answering affirmatively because other people had brought up their tanning habits, but they weren't annoyed<br>2 participants with confusion over 3, one simply did not understand, one with guilt due to the money spent on tanning<br>3 participants with confusion over 4 and answered affirmatively as they had interpreted the question as having thought about tanning in the morning in order to fit it in their schedule			

<table>
<tr><td>Derek D. Reed (2016)</td><td>Toward Quantifying the Abuse Liability of Ultraviolet Tanning: A Behavioral Economic Approach to Tanning Addiction[9]</td><td>93</td><td>Construct Validity
<table>
<tr><th></th><th>Negative mDSM-IV-TR</th><th>Positive mDSM-IV-TR</th></tr>
<tr><td>Negative mCAGE</td><td>51†</td><td>6†</td></tr>
<tr><td>Positive mCAGE</td><td>24</td><td>12</td></tr>
</table>
<table>
<tr><th></th><th>Negative mCAGE and mDSM-IV-TR</th><th>Positive mCAGE and/or mDSM-IV-TR</th></tr>
<tr><td>Never tanned</td><td>28†</td><td>0†</td></tr>
<tr><td>Tanned between 5 years and one month ago</td><td>12</td><td>16</td></tr>
<tr><td>Tanned within one month</td><td>11</td><td>26</td></tr>
</table></td><td></td></tr>
<tr><td>Solmaz P. Poorsattar (2007)</td><td>UV Light Abuse and High-Risk Tanning Behavior Among Undergraduate College Students[10]</td><td>375</td><td>Construct Validity
<table>
<tr><th></th><th>Negative mCAGE (n=329)</th><th>Positive mCAGE (n=46)</th></tr>
<tr><td>History of tanning bed burn</td><td>9%†</td><td>57%†</td></tr>
<tr><td>Family history of skin cancer</td><td>18%†</td><td>35%†</td></tr>
</table></td><td></td></tr>
<tr><td>Cecilie S. Andreassen (2018)</td><td>Tanning Addiction: Conceptualization, Assessment, and Correlates[11]</td><td>23,537</td><td>Content Validity
Tucker Lewis Index = 0.99
CFI = 0.99
Factor loadings ranged 0.78-0.91</td><td>Coefficient omega
BTAS = 0.94</td></tr>
</table>

Lisham Ashrafioun (2015)	Psychometric Assessment of the Craving to Tan Questionnaire[12]	421	**Content validity**<br><br>Eigenvalue = 3.69<br><br>**Construct Validity**	**Cronbach's alpha**<br>**TPS Obsessive =** 0.91<br><br>**TPS harmonic =** 0.89<br><br>**TPI =** 0.86<br><br>**CTQ =** 0.91

	Negative mCAGE	Positive mCAGE	Negative DSM-IV	Positive DSM-IV
**CTQ mean score (SD)**	3.1 (4.0)*	10.1 (5.5)*	2.1 (3.1)*	7.6 (5.5)*

	CTQ Pearson coefficient	Negative DSM-IV CTQ Pearson coefficient	Positive DSM-IV CTQ Pearson coefficient	Z-score, P-value	Negative mCAGE	Positive mCAGE	Z-score, P-value
**Obsessive passions**	r(419) = 0.59, p<0.001	r=0.33	r=0.51	Z = 2.42, p<0.05	r=0.44	r=0.51	Z = 0.65, p>0.05
**Harmonious passions**	r(419) = 0.55, p<0.001						
**Past month frequency of tanning**	4(419) = 0.63, p<0.001						
**Money spent on tanning in a typical month**	r(416) = 0.37, p<0.001						
**Past year frequency of problems associated with tanning**	4(419) = 0.52, p<0.001						

<table>
<tr>
<td>Lisham Ashrafioun (2014)</td>
<td>Development of a Brief Scale to Assess Frequency of Symptoms and Problems Associated with Tanning[13]</td>
<td>414</td>
<td>Construct Validity
<table>
<tr><td></td><td>Negative Tanning-DSM</td><td>Positive Tanning-DSM</td></tr>
<tr><td>TPI score mean (SD)</td><td>1.4 (2.9)*</td><td>5.6 (5.6)*</td></tr>
</table>
<table>
<tr><td></td><td>Past month tanning</td><td>Money spent on tanning</td><td>Perceived difficulty to stop tanning</td></tr>
<tr><td>TPI score</td><td>r(412) = 0.36 p=0.001</td><td>r(409) = 0.41 p=0.001</td><td>r(410) = 0.55 p=0.003</td></tr>
</table>
</td>
<td>Cronbach's alpha
CTQ = 0.86</td>
</tr>
<tr>
<td>Becirevic and Reed (2017)</td>
<td>An Initial Study of Behavioral Addiction Symptom Severity and Demand for Indoor Tanning[14]</td>
<td>80</td>
<td>Construct Validity
<table>
<tr><td></td><td>Negative BAITS (n=57)</td><td>Met one BAITS criteria (n=9)</td><td>Positive BAITS (n=14)</td></tr>
<tr><td>Geometric mean of max price</td><td>Not reported</td><td>Not reported</td><td>$12.24**</td></tr>
<tr><td>Price per session above which tanning behavior extinguishes</td><td>Not reported</td><td>Not reported</td><td>$32.81**</td></tr>
</table>
</td>
<td></td>
</tr>
<tr>
<td></td>
<td colspan="4">Design:
A total of 86 undergraduate females enrolled in an introductory psychology course and indicating use of IT in the past year participated in the study for extra credit. Participants were administered questionnaires online, and asked questions regarding hypothetical spending per month, as well as BAITS questionnaires. 6 were excluded due to non-logical responses on behavioral economics tasks.</td>
</tr>
<tr>
<td>Brenda Cartmel (2017)</td>
<td>Predictors of Tanning Dependence in White Non-Hispanic Females and Males[15]</td>
<td>499</td>
<td>Construct Validity
<table>
<tr><td></td><td>Negative on both mCAGE and mDSM-IV-TR (n=255)</td><td>Positive on both mCAGE and mDSM-IV-TR (n=122)</td></tr>
<tr><td>Lifetime indoor tanning sessions</td><td>46†</td><td>424†</td></tr>
<tr><td>Lifetime sunbathing sessions</td><td>303†</td><td>706†</td></tr>
</table>
<table>
<tr><td></td><td>Negative mCAGE</td><td>Positive mCAGE</td></tr>
<tr><td>Negative mDSM-IV-TR</td><td>255</td><td>23</td></tr>
<tr><td>Positive mDSM-IV-TR</td><td>99</td><td>122</td></tr>
</table>
</td>
<td></td>
</tr>
<tr>
<td></td>
<td colspan="4">Design:
761 non-Hispanics of European-ancestry who had previously participated in a case control study of early-onset basal cell carcinoma and had indicated willingness to be contacted were required to complete an online survey through letters sent in the mail.</td>
</tr>
</table>

Brenda Cartmel (2013)	Indoor Tanning and Tanning Dependence in Young People After a Diagnosis of Basal Cell Carcinoma[16]	178	**Construct Validity**<br>**Negative mCAGE (n=105)** / **Positive mCAGE (n=73)**<br>**Tanned indoors after BCC diagnosis**: 58%† / 38%†	
Catherine Mosher (2010)	Addiction to Indoor Tanning: Relation to Anxiety, depression, And Substance Use[17]	229	**Construct Validity**<br>For respondents who had tanned indoors: **Negative mDSM-IV-TR** / **Positive mDSM-IV-TR**<br>**Negative mCAGE**: 119† / 40†<br>**Positive mCAGE**: 20 / 50<br>**OR (95% CI) negitive on both mCAGE and DSM-IV-TR vs Positive on mCAGE and DSM-IV-TR** / **OR (95% CI) negitive on both mCAGE and DSM-IV-TR vs Positive on mCAGE or DSM-IV-TR**<br>**Frequency of indoor tanning during the past year**: 1.05 (1.03-1.07) / 1.04 (1.02-1.06)	**Cronbach's alpha mCAGE =** 0.58<br><br>**mDSM-IV-TR =** 0.56
Kimberly Miller (2018)	Prevalence of Tanning Addiction and Behavioral Health Conditions among Ethnically and Racially Diverse Adolescents[18]	2,637		**Cronbach's alpha mCAGE =** 0.55
Darren Mays (2020)	Genetic Associations with Indoor Tanning Addiction among non-Hispanic White Young Adult Women[19]	389		**Cronbach's alpha mCAGE =** 0.51<br><br>**mDSM =** 0.52<br><br>**Combined 12 item mCAGE and mDSM =** 0.69

Joel Hillhouse (2012)	Evaluating a Measure of Tanning Abuse and Dependence[20]	296	**Construct Validity**

	No diagnosis (n=248)	Tanning Abuse (n=32)	Tanning Dependence (n=16)
**Indoor tanning sessions in past 6 months mean (SD)**	4.6 (10.8)**	15.9 (10.8)**	50.6 (48.7)**
**Opiate like reactions scale mean (SD)**	8.7 (4.3)**	12.9 (4.0)**	16.3 (2.7)**

Test-Retest

	SITAD tanning dependence	SITAD tanning abuse
**Agreement 3 week interval**	97%	84%
**Reliability estimate**	phi = 0.84	phi = 0.43

<table>
<tr>
<td>Carolyn Heckman (2014)</td>
<td>Measurement of Tanning Dependence[21]</td>
<td>306</td>
<td>Content validity

CFI = 0.91

Eigen values
TAPS subscale 1: perceiving tanning as a problem = 8.36
TAPS subscale 2: opiate-like effects of tanning = 2.57
TAPS subscale 3: dissatisfaction with skin tone = 1.39
TAPS subscale 4: Tolerance of tanning = 1.07

Construct Validity
<table>
<tr><td></td><td>Negative mDSM-IV-TR</td><td>Positive mDSM-IV-TR</td><td></td></tr>
<tr><td>Negative mCAGE</td><td>233</td><td>12</td><td></td></tr>
<tr><td>Positive mCAGE</td><td>46</td><td>15</td><td>For mDSM-IV-TR and mCAGE Cohen's kappa = 0.26, indicating only fair agreement</td></tr>
</table>
<table>
<tr><td></td><td>P- value for difference in mean scores on each scale did vs did not indoor tan in the past year</td><td>P- value for difference in mean scores on each scale did vs did not indoor tan in the past month</td></tr>
<tr><td>mCAGE</td><td><0.001</td><td><0.001</td></tr>
<tr><td>mDSM-IV-TR</td><td><0.001</td><td><0.001</td></tr>
<tr><td>Taps perceiving tanning as problem subscale</td><td><0.01</td><td><0.01</td></tr>
<tr><td>Taps opiate-like effects of tanning subscale</td><td><0.001</td><td><0.001</td></tr>
<tr><td>Taps dissatisfaction with skin tone subscale</td><td><0.001</td><td><0.001</td></tr>
<tr><td>Taps tolerance to tanning subscale</td><td>> 0.05</td><td>> 0.05</td></tr>
</table>
</td>
<td>Cronbach's alpha

mCAGE = 0.64

mDSM-IV-TR = 0.59

mCAGE and mDSM-IV-TR combined = 0.7

TAPS subscale 1: perceiving tanning as a problem = 0.92

TAPS subscale 2: opiate-like effects of tanning = 0.91

TAPS subscale 3: dissatisfaction with skin tone = 0.85

TAPS subscale 4: Tolerance of tanning = 0.81</td>
</tr>
<tr>
<td>Carolyn Heckman (2008)</td>
<td>A Preliminary Investigation of the Predictors of Tanning Dependence[22]</td>
<td>400</td>
<td>Construct Validity
<table>
<tr><td></td><td>Negative mCAGE and DSM-VI-TR (n= 294)</td><td>Positive mCAGE and/or DSM-VI-TR (n= 106)</td></tr>
<tr><td>Mean (SD) lifetime indoor tanning sessions</td><td>35.93 (9)*</td><td>83.15 (10)*</td></tr>
<tr><td>Mean (SD) indoor tanning sessions last year</td><td>5.75 (2.83)*</td><td>24.20 (3.18)*</td></tr>
<tr><td>Mean (SD) hours per week sunbathing</td><td>2.44 (.35)*</td><td>7.50 (.58)*</td></tr>
</table>
</td>
<td>Cronbach's alpha
mCAGE = 0.57

DSM-IV-TR = 0.56</td>
</tr>
</table>

<table>
<tr>
<td>Katharina Diehl (2018)</td>
<td>First Evaluation of the Behavioral Addiction Indoor Tanning Screener (BAITS) in a Nationwide Representative Sample[23]</td>
<td>883</td>
<td>
Content Validity
Kaiser-Meyer-Olkin = 0.852
Bartlett < 0.001
All variables with BAITS load on one factor with a value of at least 0.662

Construct Validity
<table>
<tr><th>Agreement to item</th><th>Former tanner (n = 553)</th><th>Current tanner (n =330)</th></tr>
<tr><td>I think about indoor tanning too much.</td><td>3.80%†</td><td>10.90%†</td></tr>
<tr><td>At times I have used money intended for something else such as bills or school fees to pay for my indoor UV tanning sessions</td><td>0.40%†</td><td>3.60%†</td></tr>
<tr><td>I would continue to indoor tan, even if it meant I could spend less time on my hobbies and other interests</td><td>2.40%†</td><td>14.50%†</td></tr>
<tr><td>I would be greatly distressed if I could not indoor tan anymore</td><td>1.40%†</td><td>14.80%†</td></tr>
<tr><td>My urges to indoor tan keep getting stronger if I don't indoor tan</td><td>0.90%†</td><td>17%†</td></tr>
<tr><td>Sometimes I think about indoor tanning as soon as I wake up</td><td>0.40%†</td><td>7.20%†</td></tr>
<tr><td>It's hard for me to ignore an urge to indoor tan 56 (170) 16 (29) < 0001 Sum of 'yes' responses to BAITS items</td><td>2.90%†</td><td>17%†</td></tr>
<tr><td>Met >1 BAITS criteria</td><td>1.80%†</td><td>19.70%†</td></tr>
</table>

<table>
<tr><th>Current tanners</th><th>Negative BAITS</th><th>Positive BAITS</th></tr>
<tr><td>median (IQR) tanning sessions in the last 12 months</td><td>5.5 (9)†</td><td>20 (14)†</td></tr>
<tr><td>median (IQR) tanning sessions in the last 6 months</td><td>4 (6)†</td><td>8 (7.5)†</td></tr>
<tr><td>median (IQR) tanning sessions in the last 3 months</td><td>3 (3.4)†</td><td>6 (3)†</td></tr>
<tr><td>median (IQR) tanning sessions in the last 1 months</td><td>2 (1)†</td><td>3 (2)†</td></tr>
</table>

Face Validity

No confusion over meaning of questions in 10 cognitive interviews regarding BAITS questions
</td>
<td>Cronbach's alpha
BAITS = 0.84</td>
</tr>
<tr>
<td></td>
<td colspan="4">Secondary Analysis:
Ten cognitive interviews with tanning bed users were carried out. The aim of these interviews was to test if the seven BAITS items were intelligible and interpreted by the participants as intended. Six of the 10 participants were women. Five were current users (use within the past 12 months) and five former users of tanning beds (last use more than 12 months ago). Each interview was around one hour and carried out by a trained cognitive interviewer using different probing techniques to scrutinize answers including comprehension.</td>
</tr>
</table>

Kelsey Nogg (2018)	Correlates of Tanning Dependence Among Sexual Minority Males[24]	230	**Construct Validity**			**KR-20**<br>**BAITS = 0.83**
				**BAITS outcome binomial regression (SE)**		
			**Intent to Tan**	b = 0.90 (0.14) ‡		
			**Frequency of indoor tanning**	b = 0.75 (0.15) ‡		
Smita Banerjee (2014)	Indoor tanning addiction tendencies: Role of positive tanning beliefs, perceived vulnerability, and tanning risk knowledge[25]	551	**Construct Validity**			
				**OR positive mCAGE (95% CI)**	**Wald**	
			**Frequency of indoor tanning**	1.37 (1.10 – 1.69	7.99	
			**Indoor Tanning use Intention (3-months)**	2.11 (1.42 – 3.16)	13.35	
Suzeanne Benet (2008)	Addiction as an indicator of vulnerability: The case of indoor tanning[26]	102	**Construct Validity**			
				**Negative mCAGE**	**Positive mCAGE**	
			**Expected times indoor tanning in next 6 months**	16.2*	11.2*	

* Statistically significant P-value t-test
\# Statistically significant P-value likelihood ratio
† Statistically significant P-value chi-squared
‡ Statistically significant P-value for linearity
** Statistically significant P-value ANOVA

References

1. Ashrafioun L, Bonar EE: Tanning addiction and psychopathology: Further evaluation of anxiety disorders and substance abuse. J Am Acad Dermatol. 2014, 70:473-480. 10.1016/j.jaad.2013.10.057
2. Adinoff B, Aubert P, Price J, et al.: Dopamine efflux in response to ultraviolet radiation in addicted sunbed users. Neuropsychopharmacology. 2014, 39:S191-S192. 10.1038/npp.2014.280
3. Venning VL, Fernandez-Penas P: Nothing healthy about a TAN-increasing prevalence of tanning addiction. Journal of the Dermatology Nurses' Association. 2020, 12.
4. Warthan MM, Uchida T, Wagner RF, Jr.: UV light tanning as a type of substance-related disorder. Arch Dermatol. 2005, 141:963-966. 10.1001/archderm.141.8.963
5. Toledo A, Yli-Uotila E, Kautiainen H, Pirkola S, Partonen T, Snellman E: Tanning dependence and seasonal affective disorder are frequent among sunbathers but are not associated. Psychiatry Res. 2019, 272:387-391. 10.1016/j.psychres.2018.12.090
6. Stawczyk M, Łakis A, Ulatowska A, Szczerkowska-Dobosz A: Evaluation of risk of tanning addiction in a selected population of women aged 15-30. Przeglad Dermatologiczny. 2011, 98:305-311.
7. Stapleton JL, Hillhouse JJ, Turrisi R, Baker K, Manne SL, Coups EJ: The Behavioral Addiction Indoor Tanning Screener (BAITS): An Evaluation of a Brief Measure of Behavioral Addictive Symptoms. Acta Derm Venereol. 2016, 96:552-553. 10.2340/00015555-2290
8. Schneider S, Schirmbeck F, Bock C, Greinert R, Breitbart EW, Diehl K: Casting shadows on the prevalence of tanning dependence: an assessment of mCAGE criteria. Acta dermato-venereologica. 2015, 95:162-168. 10.2340/00015555-1907
9. Reed DD, Kaplan BA, Becirevic A, Roma PG, Hursh SR: Toward quantifying the abuse liability of ultraviolet tanning: A behavioral economic approach to tanning addiction. J Exp Anal Behav. 2016, 106:93-106. 10.1002/jeab.216
10. Poorsattar SP, Hornung RL: UV light abuse and high-risk tanning behavior among undergraduate college students. Journal of the American Academy of Dermatology. 2007, 56:375-379. 10.1016/j.jaad.2006.08.064
11. Andreassen CS, Pallesen S, Torsheim T, Demetrovics Z, Griffiths MD: Tanning addiction: conceptualization, assessment and correlates. Br J Dermatol. 2018, 179:345-352. 10.1111/bjd.16480
12. Ashrafioun L, Bonar EE: Psychometric assessment of the craving to tan questionnaire. Am J Drug Alcohol Abuse. 2015, 41:74-81. 10.3109/00952990.2014.939754
13. Ashrafioun L, Bonar EE: Development of a brief scale to assess frequency of symptoms and problems associated with tanning. J Am Acad Dermatol. 2014, 70:588-589. 10.1016/j.jaad.2013.11.041
14. Becirevic A, Reed DD, Amlung M, Murphy JG, Stapleton JL, Hillhouse JJ: An initial study of behavioral addiction symptom severity and demand for indoor tanning. Exp Clin Psychopharmacol. 2017, 25:346-352. 10.1037/pha0000146
15. Cartmel B, Bale AE, Mayne ST, et al.: Predictors of tanning dependence in white non-Hispanic females and males. Journal of the European Academy of Dermatology and Venereology. 2017, 31:1223-1228. 10.1111/jdv.14138
16. Cartmel B, Ferrucci LM, Spain P, et al.: Indoor tanning and tanning dependence in young people after a diagnosis of basal cell carcinoma. JAMA Dermatol. 2013, 149:1110-1111. 10.1001/jamadermatol.2013.5104
17. Mosher CE, Danoff-Burg S: Addiction to indoor tanning: relation to anxiety, depression, and substance use. Arch Dermatol. 2010, 146:412-417. 10.1001/archdermatol.2009.385
18. Miller KA, Piombo SE, Cho J, et al.: Prevalence of Tanning Addiction and Behavioral Health Conditions among Ethnically and Racially Diverse Adolescents. J Invest Dermatol. 2018, 138:1511-1517. 10.1016/j.jid.2018.02.018
19. Mays D, Ahn J, Zhang B, Atkins MB, Goerlitz D, Tercyak KP: Genetic Associations with Indoor Tanning Addiction among non-Hispanic White Young Adult Women. Ann Behav Med. 2020, 54:1-9. 10.1093/abm/kaz021

20. Hillhouse JJ, Baker MK, Turrisi R, et al.: Evaluating a measure of tanning abuse and dependence. Arch Dermatol. 2012, 148:815-819. 10.1001/archdermatol.2011.2929
21. Heckman CJ, Darlow S, Kloss JD, et al.: Measurement of tanning dependence. J Eur Acad Dermatol Venereol. 2014, 28:1179-1185. 10.1111/jdv.12243
22. Heckman CJ, Egleston BL, Wilson DB, Ingersoll KS: A preliminary investigation of the predictors of tanning dependence. Am J Health Behav. 2008, 32:451-464. 10.5555/ajhb.2008.32.5.451
23. Diehl K, Görig T, Breitbart EW, et al.: First evaluation of the Behavioral Addiction Indoor Tanning Screener (BAITS) in a nationwide representative sample. British Journal of Dermatology. 2018, 178:176-182. 10.1111/bjd.15888
24. Nogg KA, Lamb KM, Rooney BM, Blashill AJ: Correlates of tanning dependence among sexual minority males. Psychology of Men & Masculinities. 2019, 20:208-213. 10.1037/men0000167
25. Banerjee SC, Hay JL, Greene K: Indoor tanning addiction tendencies: Role of positive tanning beliefs, perceived vulnerability, and tanning risk knowledge. Addiction Research & Theory. 2015, 23:156-162. 10.3109/16066359.2014.955479
26. Benet SB, Kraft FB: Addiction as an indicator of vulnerability: The case of indoor tanning. Journal of Consumer Behaviour. 2019, 18:378-386. 10.1002/cb.1777
